# Genome Sequence of a Pathogenic Vibrio cholerae O1 El Tor Strain Defective for the Entire *Vibrio* Pathogenicity Island 1, Isolated in Eastern Democratic Republic of the Congo

**DOI:** 10.1128/MRA.00454-20

**Published:** 2020-06-25

**Authors:** Leonid M. Irenge, Jean-François Durant, Jérôme Ambroise, Prudence N. Mitangala, Bertrand Bearzatto, Jean-Luc Gala

**Affiliations:** a Center for Applied Molecular Technologies, Institute of Clinical and Experimental Research, Université Catholique de Louvain, Brussels, Belgium; b Defence Laboratories Department, ACOS Ops & Trg, Belgian Armed Forces, Peutie, Belgium; c Laboratoire Provincial du Nord-Kivu, Goma, Democratic Republic of the Congo; Broad Institute

## Abstract

We report here a complete genome sequence of a Vibrio cholerae O1 El Tor (Inaba; sequence type 515 [ST515]) strain isolated from a cholera patient in North Kivu Province, Democratic Republic of the Congo (DRC), which showed a complete deletion (∼80 kb) of the *Vibrio* pathogenicity island 1.

## ANNOUNCEMENT

**FIG 1:**
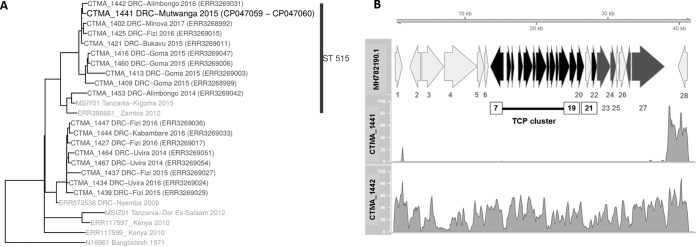
(A) An SNP-based phylogenomic tree of representative isolates of ST69 and ST515, including the CTMA_1441 isolate, was built using kSNP v3.1 (11). The 7PET V. cholerae O1 biotype El Tor 216 N19691 belonging to wave 1 was used as the outgroup. (B) Coverage plots of two V. cholerae strains (CTMA_1441 and CTMA_1442) obtained from a mapping of Illumina reads against the VPI-1 genomic sequence (GenBank accession number MH782190.1) using minimap2 (7). Genes belonging to the TCP cluster are shown with black arrows and annotated according to data in GenBank. The complete list of genes includes putative transposase (QCB64826.1) (1), *aldA* (2), *tagA* (3), putative inner membrane protein (QCB64829.1) (4), putative zinc metalloprotease (QCB64830.1) (5), TagD (6), TCP (I, P, H, A, B, Q, C, R, D, S, T, E, and F) (7 to 19), *toxT* (20), *tcpJ* (21), *acfB* (22), *acfC* (23), hypothetical protein (QCB64849.1) (24), *tagE* (25), *acfA* (26), *acfD* (27), and *int* (28).

Vibrio cholerae, the causative agent of the pandemic human disease cholera (1), is responsible for successive cholera outbreaks in the Democratic Republic of the Congo (DRC) (2, 3). Whole-genome sequencing (WGS) from a large series of clinical isolates of V. cholerae was recently reported (4), most of them from the seventh pandemic V. cholerae O1 El Tor (7PET) lineage, T10 sublineage. They clustered in two sequence type 69 (ST69) and ST515 multilocus sequence typing (MLST) subclades. We report here the genome sequence of a new V. cholerae O1 El Tor isolate, CTMA-1441, belonging to the ST515 cluster, which was isolated from a cholera patient in Mutwanga, Beni Territory, North-Kivu, DRC (Fig. 1A).

A rectal swab sample was taken after obtaining the patient's oral informed consent (given his low level of literacy), and ethical approval to conduct the study as reported previously (4). The swab was incubated in saline and alkaline peptone water broth for 6 h and subsequently streaked onto thiosulfate-citrate-bile salts sucrose (TCBS) agar at 37°C for 16 to 24 h, and DNA was extracted using the phenol chloroform method. The short-read whole-genome sequence (WGS) libraries were prepared from 1 ng DNA using a Nextera XT DNA library preparation kit (Illumina, San Diego, CA, USA). This library was then paired-end (2 × 300 bp) sequenced on a MiSeq platform (Illumina), generating 2 × 391,193 reads. The long reads were generated using MinION sequencing (Oxford Nanopore Technologies, UK). A library was prepared from 400 ng of genomic DNA using a rapid barcoding sequencing kit (SQK-RBK004) and sequenced in a FLO-MIN106 (R9.4.1) flow cell for a 48-h run. Fast5 files were then base called on the MinIT instrument using default settings in MinKNOW v18.12 and Guppy v3.0.3 and a high-accuracy (HAC) flip-flop methodology. This generated 209,798 reads with an average read length of 6.02 kb. All reads were quality checked using FastQC (http://www.bioinformatics.babraham.ac.uk/projects/fastqc/) and assembled *de novo* using SPAdes v3.13.1 (http://cab.spbu.ru/software/spades/) (5) with default settings. Sequences were assembled in 4 contigs, and we manually finished sequence gaps using PCR amplification and Sanger sequencing (ABI 373 sequencer).

The complete genome has a total size of 4,042,777 bp with coverages of 58× and 312× (with Illumina and MinION data, respectively) and consists of two chromosomes (2,983,103 and 1,059,674 bp for the large and small chromosomes, respectively). The DNA G+C contents were calculated at 47.8% and 46.9% for the large and small chromosomes, respectively. Genome annotation was done using the Prokaryotic Genome Annotation Pipeline (PGAP) v4.10 (6). PGAP annotation identified a total of 3,613 coding DNA sequences, 25 rRNA sequences (9 5S, 8 16S, and 8 23S rRNA sequences), and 100 tRNA sequences. A deletion (∼80 kb) extending from the VC_0774 to VC_0845 loci was identified by comparing the CTMA-1441 and N16961 genomes and subsequently verified by a mapping of Illumina reads against the *Vibrio* pathogenicity island 1 (VPI-1) genomic sequence using minimap2 (7) (Fig. 1B). Considering the subsequent loss of the entire VPI-1, including the toxin coregulated pilus (TCP) cluster that plays a critical role in the colonization of the host gut mucosal layer (8), one would expect this large deletion to affect the virulence and transmission of VPI-1-defective isolates. Interestingly, and despite their extreme scarcity, few data suggest, however, that partial or even total deletion of VPI-1 virulence genes affects the emergence of cholera disease in 7PET strains, as was also the case with our strain (9, 10). The complete genome of the V. cholerae strain CTMA-1441 therefore sheds light on the extent of genetic variability of V. cholerae isolates from eastern DRC. The choleragenic property of this isolate deserves further consideration regarding the association of cholera endemicity and cross-border epidemic outbreaks in this region.

### Data availability.

This whole-genome sequence comprising the large and small chromosomes has been deposited at DDBJ/ENA/GenBank under accession numbers CP047059 and CP047060. The raw sequence reads have been deposited in the NCBI Sequence Read Archive under accession numbers SRX8230516 and SRX8230515.
